# Plasma Chemokines in Patients with Alcohol Use Disorders: Association of CCL11 (Eotaxin-1) with Psychiatric Comorbidity

**DOI:** 10.3389/fpsyt.2016.00214

**Published:** 2017-01-18

**Authors:** Nuria García-Marchena, Pedro Fernando Araos, Vicente Barrios, Laura Sánchez-Marín, Julie A. Chowen, María Pedraz, Estela Castilla-Ortega, Pablo Romero-Sanchiz, Guillermo Ponce, Ana L. Gavito, Juan Decara, Daniel Silva, Marta Torrens, Jesús Argente, Gabriel Rubio, Antonia Serrano, Fernando Rodríguez de Fonseca, Francisco Javier Pavón

**Affiliations:** ^1^Unidad Gestión Clínica de Salud Mental, Instituto de Investigación Biomédica de Málaga (IBIMA), Hospital Regional Universitario de Málaga, Málaga, Spain; ^2^Facultad de Psicología, Universidad Complutense de Madrid, Madrid, Spain; ^3^Department of Endocrinology, Hospital Infantil Universitario Niño Jesús, Madrid, Spain; ^4^Department of Pediatrics, Universidad Autónoma de Madrid, Madrid, Spain; ^5^CIBER Fisiopatología de la obesidad y nutrición (CIBERobn), Instituto de Salud Carlos III, Madrid, Spain; ^6^Servicio de Psiquiatría, Hospital Universitario 12 de Octubre, Madrid, Spain; ^7^Institut de Neuropsiquiatria i Addiccions (INAD), Barcelona, Spain; ^8^Institut Hospital del Mar d’Investigacions Mèdiques (IMIM), Barcelona, Spain; ^9^Department of Psychiatry, Universitat Autònoma de Barcelona (UAB), Barcelona, Spain

**Keywords:** chemokine, alcohol use disorder, psychiatric comorbidity, outpatient setting, PRISM, eotaxin, sex

## Abstract

Recent studies have linked changes in peripheral chemokine concentrations to the presence of both addictive behaviors and psychiatric disorders. The present study further explore this link by analyzing the potential association of psychiatry comorbidity with alterations in the concentrations of circulating plasma chemokine in patients of both sexes diagnosed with alcohol use disorders (AUD). To this end, 85 abstinent subjects with AUD from an outpatient setting and 55 healthy subjects were evaluated for substance and mental disorders. Plasma samples were obtained to quantify chemokine concentrations [C–C motif (CC), C–X–C motif (CXC), and C–X_3_–C motif (CX_3_C) chemokines]. Abstinent AUD patients displayed a high prevalence of comorbid mental disorders (72%) and other substance use disorders (45%). Plasma concentrations of chemokines CXCL12/stromal cell-derived factor-1 (*p* < 0.001) and CX_3_CL1/fractalkine (*p* < 0.05) were lower in AUD patients compared to controls, whereas CCL11/eotaxin-1 concentrations were strongly decreased in female AUD patients (*p* < 0.001). In the alcohol group, CXCL8 concentrations were increased in patients with liver and pancreas diseases and there was a significant correlation to aspartate transaminase (*r* = +0.456, *p* < 0.001) and gamma-glutamyltransferase (*r* = +0.647, *p* < 0.001). Focusing on comorbid psychiatric disorders, we distinguish between patients with additional mental disorders (*N* = 61) and other substance use disorders (*N* = 38). Only CCL11 concentrations were found to be altered in AUD patients diagnosed with mental disorders (*p* < 0.01) with a strong main effect of sex. Thus, patients with mood disorders (*N* = 42) and/or anxiety (*N* = 16) had lower CCL11 concentrations than non-comorbid patients being more evident in women. The alcohol-induced alterations in circulating chemokines were also explored in preclinical models of alcohol use with male Wistar rats. Rats exposed to repeated ethanol (3 g/kg, gavage) had lower CXCL12 (*p* < 0.01) concentrations and higher CCL11 concentrations (*p* < 0.001) relative to vehicle-treated rats. Additionally, the increased CCL11 concentrations in rats exposed to ethanol were enhanced by the prior exposure to restraint stress (*p* < 0.01). Concordantly, acute ethanol exposure induced changes in CXCL12, CX_3_CL1, and CCL11 in the same direction to repeated exposure. These results clearly indicate a contribution of specific chemokines to the phenotype of AUD and a strong effect of sex, revealing a link of CCL11 to alcohol and anxiety/stress.

## Introduction

Substantial evidence suggests that the immune system modulates behaviors through specific actions of inflammatory signals in the central nervous system (CNS) (1). Among these signals, chemokines are chemoattractants involved in cellular migration and intercellular communication (2), which are known to regulate neuronal development, survival, and regeneration in the CNS (3). Chemokines might reach the brain through the blood compartment, but also they can be released by neurons and glial cells in response to physiological or pathological conditions. In particular, the resident immune cells (microglia) have a prominent role in the mediation of chemokine actions. They have been reported to be involved in synaptic remodeling, an essential step in behavioral consolidation and acquisition. Moreover, chemokine receptors expressed by microglia contribute to brain development, especially during critical developmental periods, controlling essential processes such as synaptic pruning (4, 5). Consequently, dysregulation in the chemokine signaling and neuroinflammation have been proposed to contribute to cognitive dysfunctions and mental diseases (1).

Additionally, alterations in circulating chemokines {e.g., chemokine C–C motif ligand 2 [CCL2, also referred to monocyte chemoattractant protein-1 (MCP-1)], chemokine C–C motif ligand 11 [CCL11, eotaxin-1], chemokine C–X–C motif ligand 8 [CXCL8, interleukin-8 (IL-8)], and chemokine C–X–C motif ligand 12 [CXCL12, stromal cell-derived factor-1 (SDF-1)]} have been recently associated with psychiatric disorders such as cocaine use disorders (6), mood disorders (7, 8), generalized anxiety (9), personality disorders (9), and Alzheimer’s disease (10). Collectively, these findings suggest that these inflammatory signals could be considered as pathologically relevant biomarkers or therapeutic targets for psychopathologies (11).

Concerning substance use disorders, a growing literature indicates that the pharmacodynamic actions of alcohol and other drugs of abuse involve the central activation of immune signaling (12). Thus, studies in rodents have revealed that drug-induced alterations in chemokines, such as CXCL12 and CCL2, promote common addictive behaviors in a brain region-specific manner (2, 13–15). Additional studies in alcoholics have reported activated microglia and increased concentrations of CCL2 and other inflammatory molecules in multiple brain regions (3, 16). These elevated CCL2 concentrations have also been detected in the cerebrospinal fluid of alcoholics, correlating with plasma concentrations of liver transaminases (17). Chemokines and their receptors are known for their role in liver injury, especially the CC chemokine family in animal models (18), and both alcoholic hepatitis and cirrhosis are associated with distinct patterns of chemokine expression such as CCL8, CCL2, chemokine C-C motif ligand 3 [CCL3, also referred to macrophage inflammatory protein-1 alpha (MIP-1α)], and chemokine C-C motif ligand 4 (CCL4, macrophage inflammatory protein-1 beta) (19).

These findings confirm the existence of a close interaction between inflammatory signals from peripheral tissues (e.g., liver) and neuroinflammation through the blood stream and the blood–brain barrier (20). In line with these observations, we reported recently that plasma levels of CXCL12 and chemokine C–X_3_–C motif ligand 1 (CX_3_CL1, also referred to fractalkine) are correlated to cocaine symptom severity, which allows stratifying the cocaine addicts in patients with different incidence of comorbid psychiatric disorders (6, 21). However, in the case of alcohol use disorders (AUD), plasma chemokines have been evaluated almost exclusively in the context of alcoholic hepatitis (18, 22). Taking into account all these observations, it is feasible to hypothesize that the chronic use of drugs of abuse leads to progressive changes in the neurobiology and behavior that might be mediated not only by the innate immune system, but also by the coordinated actions of chemokines. The impact of these chemokine-mediated responses in the brain might contribute to the addicted phenotype and to the high prevalence of psychopathologies observed in addicts (3).

In the present study, we tested the hypothesis that lifetime AUD are associated with altered circulating chemokine concentrations that could be observed during abstinence in male and female patients. Furthermore, variables associated with alcohol addiction (i.e., abstinence, liver and/or pancreas diseases, and criteria for AUD) and psychiatric comorbidity (other substance use disorders and mental disorders) could be associated with alterations in the expression of certain chemokines in the plasma of patients with AUD in outpatient treatment. Additional studies in rats were performed to test the impact of alcohol administration on the chemokine expression, exploring also the combined association of stress and alcohol exposure.

## Materials and Methods

### Participants and Recruitment

The present cross-sectional study was performed in a total of 140 White Caucasian subjects who were divided into alcohol and control groups. Eighty-five abstinent subjects diagnosed with AUD (alcohol abuse or dependence) were recruited from outpatient programs for alcohol use disorder at *Hospital Universitario 12 de Octubre* in Madrid (Spain) for a period of 18 months (November 2013–May 2015). Fifty-five healthy individuals with no history of substance use disorders or pathological use of substances were matched with the alcohol group for age, sex ratio, and body mass index (BMI). The average participant was a man (65%) of 46.6 years and a BMI of 25.5 kg/m^2^.

The participants in the alcohol group had to meet eligibility criteria based on (A) *inclusion criteria*: age ≥18 years up to 60 years of age, lifetime AUD, and at least 4 weeks of abstinence before testing; (B) *exclusion criteria*: presence of infectious diseases, incapacitating cognitive alterations to complete psychiatric interviews, and pregnancy for female participants. A breathalyzer was used daily for estimating blood alcohol content from a breath sample. Because of high incidence of alcohol-induced diseases in accessory organs (liver and pancreas), we included these individuals in the alcohol group. A description of the sample is presented in Table 1.

**Table 1:** **Baseline sociodemographic characteristics in abstinent alcohol use disorders patients and controls**.

Variable		Group		
		Alcohol (*N* = 85)	Control (*N* = 55)	*p*-Value
Age [mean (SD)]	Years	47.16 (7.27)	45.20 (9.97)	0.211^a^
BMI [mean (SD)]	kg/m^2^	25.69 (3.86)	24.82 (3.38)	0.177^a^
Sex [*N* (%)]	Women	27 (31.8)	21 (38.2)	0.469^b^
	Men	58 (68.2)	34 (61.8)	
Marital status [*N* (%)]	Single	28 (32.9)	26 (47.3)	**0.012^b^**
	Married/cohabiting	27 (31.8)	22 (40.0)	
	Divorced/separated/widowed	30 (35.3)	7 (12.7)	
Education [*N* (%)]	≤Primary/elementary	24 (28.2)	10 (18.2)	0.227^b^
	≥Secondary	61 (71.8)	45 (81.8)	
Occupation [*N* (%)]	Employed	47 (55.3)	42 (76.4)	**0.012^b^**
	Unemployed	38 (44.7)	13 (23.6)	
Psychological/psychiatric support [*N* (%)]	No	0 (0.0)	52 (94.5)	**<0.001^b^**
	Yes	85 (100)	3 (5.5)	

*BMI, body mass index*.

*Bold numbers denote significant differences (*p* < 0.05)*.

*^a^p-Value from Student’s t-test*.

*^b^p-Value from Fisher’s exact test or chi-square test*.

Alcohol use disorder patients were being treated with pharmacological approaches and psychosocial techniques. Sixty-one subjects were treated with disulfiram during the last year. Regarding psychiatric medication, 60 patients were treated with psychiatric medication during the last year: antidepressants (*N* = 35), anxiolytics (*N* = 26), anticonvulsants (*N* = 29), and antipsychotics (*N* = 7).

### Ethics Statement

Written informed consent was obtained from each participant after a complete description of the study and discussing any question or issue. The study and protocols for recruitment were approved by the Ethics Committee of the *CEI Provincial de Málaga* and *Hospital Universitario 12 de Octubre* in accordance with the “Ethical Principles for Medical Research Involving Human Subjects” adopted in the Declaration of Helsinki by the World Medical Association (64th WMA General Assembly, Fortaleza, Brazil, October 2013), Recommendation *No. R (97) 5* of the Committee of Ministers to Member States on the Protection of Medical Data (1997), and Spanish data protection act (*Ley Orgánica 15/1999 de Protección de Datos, LOPD*).

### Clinical Assessments

Patients from outpatient programs for alcohol use disorder were evaluated according to “Diagnostic and Statistical Manual of Mental Disorders-4th Edition-Text Revision” (DSM-IV-TR) criteria, using the Spanish version of the “Psychiatric Research Interview for Substance and Mental Diseases” (PRISM) (23). Diagnoses were made using two time-frames: “current” (criteria were met within the past year) and “past” (criteria were met before the previous 12 months). Lifetime prevalence, taking into accounts both current and past diagnoses, was used to present the frequency of substance use disorders and other mental disorders. DSM-IV-TR criteria for substance dependence and abuse were used to diagnose substance use disorders and determine the severity of substance use disorders (6, 24).

Control subjects were initially evaluated by PRISM (for substance screening and abuse and dependence) and subsequently by the Spanish version of the “Composite International Diagnostic Interview” (CIDI) to detect psychiatric disorders (25). All the interviews were performed by experienced psychologists who had received both PRISM and CIDI training.

### Collection of Plasma Samples

Blood samples were obtained in the morning (9:00–11:00 a.m.) after fasting for 8–12 h (previous to the psychiatric interviews). Venous blood was extracted into 10 mL K_2_-EDTA tubes (BD, Franklin Lakes, NJ, USA) and was immediately processed to obtain plasma. Blood samples were centrifuged at 2,200 × *g* for 15 min (4°C) and individually assayed for detecting infectious diseases by three rapid tests for HIV (HIVTOP^®^), hepatitis B (HBVTOP^®^), and hepatitis C (HCVTOP^®^) purchased from ALL.DIAG (Strasbourg Cedex, France). Samples displaying infection were discarded following safety protocols. Plasma samples were individually characterized, registered, and stored at −80°C until further analyses.

### Multiplex Immunoassays

Chemokines were chosen based on previous studies about inflammatory mediators and psychiatric disorders and addiction (6). A Bio-Plex Suspension Array System 200 (Bio-Rad Laboratories, Hercules, CA, USA) was used to quantify chemokine concentrations in plasma with a Procarta Immunoassay Kit using polystyrene beads and an appropriate diluent Plasma Standard Diluent Kit (Affymetrix-Panomics, Santa Clara, CA, USA). This method of analysis is based on the Luminex technology and a human chemokine 6-plex panel was used to simultaneously detect the following analytes: CXCL8 (IL-8), CXCL12 (SDF-1), CX_3_CL1 (fractalkine), CCL2 (MCP-1), CCL3 (MIP-1α), and CCL11 (eotaxin-1). The measurements of these analytes in plasma were performed following the manufacturer’s instructions (6). Raw data were analyzed using the Bio-Plex Manager Software 4.1 (Bio-Rad Laboratories, Hercules, CA, USA). Data are expressed as picograms of protein per milliliter of plasma.

### Determination of Biochemical Parameters Related to Hepatic and Pancreatic Functions

In addition to immunoassays, plasma samples of the participants were assessed for markers of hepatic and pancreatic functions in a clinical analysis laboratory (Analisis Clinicos Rodriguez Vergara S.L., Malaga, Spain). We examined the following parameters: aspartate transaminase (AST/GOT), alanine transaminase (ALT/GPT), gamma-glutamyltransferase (GGT), pancreatic α-amylase, and pancreatic lipase. The reference ranges from the laboratory were established as follows: 0–40, 0–40, 0–45, 5–100, and 0–67 IU/L, respectively.

### Animals and Ethics Statements

Animal studies were conducted on 5- to 8-week-old male Wistar rats (Charles River Laboratories España S.A., Barcelona, Spain) weighing 200–250 g at the beginning of the experiments. Rats were kept in clear plastic cages under a 12-h light/dark cycle (lights off at 8:00 p.m.) in a room at ambient temperature (23°C) and humidity (55%) in the Animal Resource Center at the University of Málaga (Spain). Unless otherwise indicated, water and chow pellets were available *ad libitum* throughout the course of these studies.

Experiments and procedures were conducted under strict adherence to the European Directive 2010/63/EU on the protection of animals used for scientific purposes and with Spanish regulations (RD 53/2013 and 178/2004). All efforts were made to minimize unnecessary suffering. All protocols were approved by the Committee on the Ethics of Universidad de Málaga (CEUMA, 7-2016-A).

### Experimental Procedures

For ethanol experiments, rats were accustomed to the experimental conditions including handling and intragastric gavage (i.g.) procedure for a period of 1 week using saline. The number of animals for each experimental group was ranged 7–8.

#### Repeated Ethanol Exposure

Rats were gavaged daily with a solution of ethanol (3 g/kg in saline) or saline as vehicle during 4 weeks. An additional group of rats were individually restrained for a period of 90 min in a Plexiglas cylindrical restrainer (diameter × length: 6 cm × 20 cm) with breathing holes in a separate testing room 48 h before beginning the ethanol or saline i.g. treatment. All rats were sacrificed by decapitation 1 week after the last ethanol or saline i.g. session in a room provided with low-intensity white noise.

#### Acute Ethanol Exposure

Another set of rats were acutely treated with a solution of ethanol (3 g/kg in saline, i.g.) and returned to the home cages immediately. Rats were sacrificed by decapitation at 30, 60, 120, and 240 min after the ethanol administration in a room provided with low-intensity white noise. To evaluate the effect of time on chemokine expression, two groups of naive rats were sacrificed at the beginning (*t* = 0 min) and at the end (*t* = 240 min) of the experiment with no ethanol exposure.

### Immunoassay Analyses

Blood samples from the ethanol experiments in rats were immediately collected and centrifuged (2,000 × *g* for 15 min) and plasmas were kept at −80°C for immunoassay analyses. CXCL12 (SDF-1), CX_3_CL1 (fractalkine), and CCL11 (eotaxin-1) were measured in rat plasma with commercial enzyme-linked immunosorbent assay (ELISA) kits in 96-well plate format following the manufacturer’s instructions: ELISA Kit for SDF-1 (product #: SEA122Ra; Cloud-Clone Corp., Wuhan, Hubei, PR China), Rat Fractalkine ELISA Kit (CX_3_CL1) (product #: ab100760; Abcam, Cambridge, UK), and Rat Eotaxin 1 (Eotaxin 1/CCL11/ECF) ELISA Kit (product #: CSB-E07319r; Cusabio, College Park, MD, USA). The plates were read at 450 nm within 30 min at a set wavelength of 540 nm or 570 nm. Data are expressed as nanograms of protein per milliliter of plasma.

### Statistical Analyses

All clinical data in the tables are expressed as number and percentage of subjects [*N* (%)] or mean and SD of concentrations [mean (SD)]. The significance of differences in categorical and normal continuous variables was determined using Fisher’s exact test (chi-square test) and Student’s *t*-test, respectively.

Statistical analysis of chemokine concentrations was performed using multiple analysis of covariance (ANCOVA) to indicate the relative effect of explanatory variables and their interactions on the chemokine expression in the plasma, controlling for additional covariates. Log(10)-transformation was used to ensure statistical assumptions for positive skewed distributions. The *post hoc* comparisons in ANCOVA were performed using Sidak’s correction test. Estimated marginal means [95% confidence intervals (95% CI)] of chemokine concentrations were expressed and represented in the figures after back-transformation if that was the case. In addition, correlation analyses were performed using the Pearson’s coefficient (*r*). Regarding data from rat studies, Student’s *t*-test and analysis of variance (ANOVA) with multiple *post hoc* tests (Dunnett and Tukey tests) were conducted for statistical analyses and chemokine concentrations in rat plasma are expressed as mean and SEM.

A *p*-value < 0.05 was considered statistically significant. Statistical analyses were performed using the GraphPad Prism version 5.04 software (GraphPad Software, San Diego, CA, USA) and IBM SPSS Statistical version 22 software (IBM, Armonk, NY, USA).

## Results

### Plasma Chemokine Concentrations in Abstinent AUD Patients and Controls

**Figure 1:**
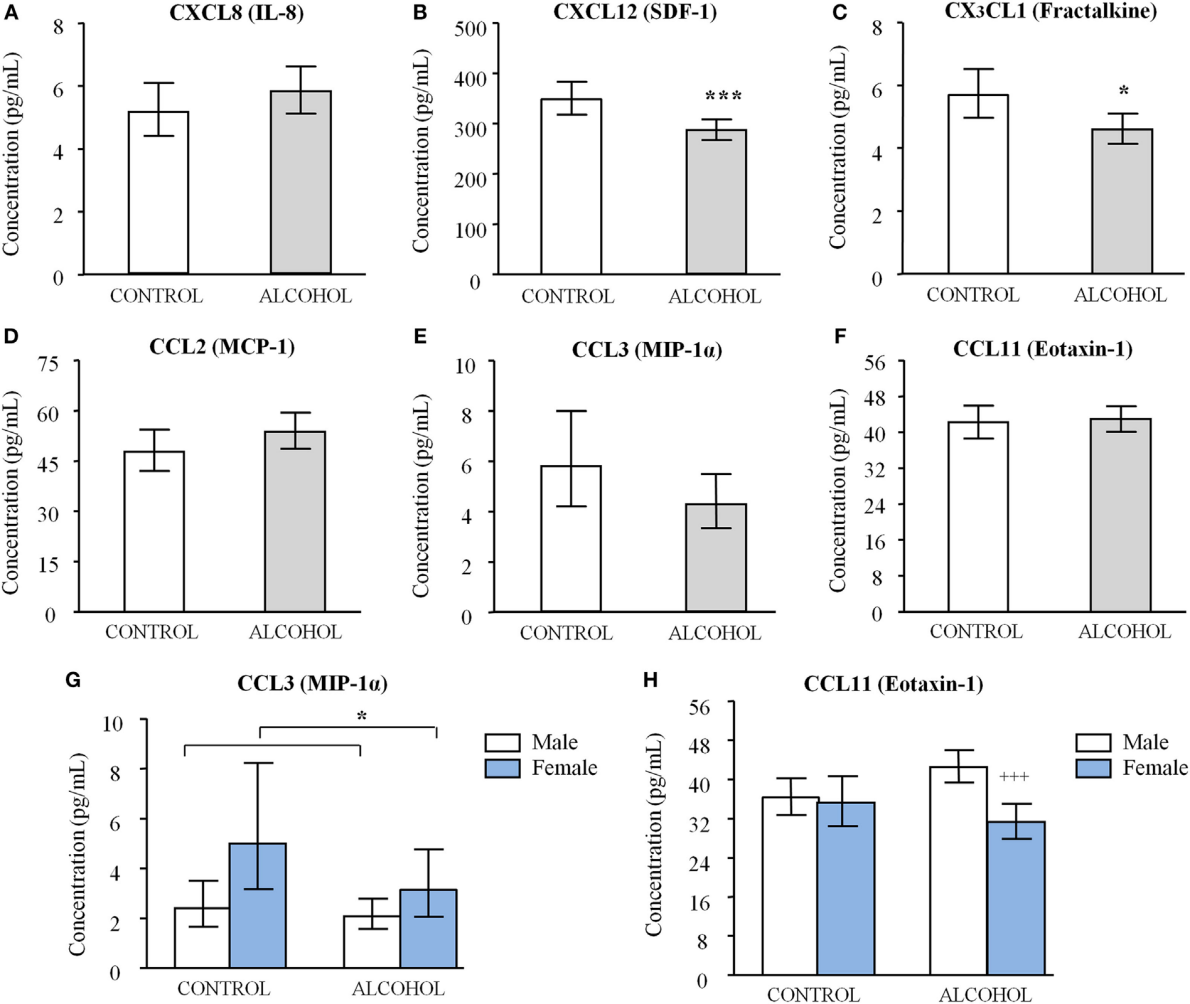
**Plasma chemokine concentrations in abstinent alcohol use disorders (AUD) patients and control subjects**. **(A)** CXCL8 [interleukin-8 (IL-8)]; **(B)** CXCL12 [stromal cell-derived factor-1 (SDF-1)]; **(C)** CX_3_CL1 (fractalkine); **(D)** CCL2 [monocyte chemoattractant protein-1 (MCP-1)]; **(E)** CCL3 [macrophage inflammatory protein-1 alpha (MIP-1α)]; and **(F)** CCL11 (eotaxin-1) concentrations according to “history of AUD.” Bars are estimated marginal means and 95% confidence intervals (95% CI) (picograms per milliliter). Data were analyzed by two-way analysis of covariance (ANCOVA) and *p < 0.05 and ***p < 0.001 denote a significant main effect of “history of AUD.” **(G)** CCL3 (MIP-1α) and **(H)** CCL11 (eotaxin-1) concentrations according to “history of AUD” and “sex.” Bars are marginal means and 95% CI (picograms per milliliter). Data were analyzed by two-way ANCOVA and *p < 0.05 denotes a significant main effect of “sex.” ^+++^*p* < 0.001 denotes significant differences compared to male AUD patients because there was an interaction of factors.

The impact of lifetime diagnosis of AUD on the plasma levels of chemokines in men and women was investigated using a two-way ANCOVA with “history of AUD” and “sex” (*N* = 92 men and *N* = 48 women) as factors, and “age” and “BMI” as covariates. Concentrations were examined to ensure that statistical assumptions were met and a base-10 logarithmic transformation of chemokine concentrations was conducted for CXCL8, CXCL12, CX_3_CL1, CCL2, and CCL3. Estimated marginal means for “history of AUD” were represented after back-transformation in Figure 1.

Plasma concentrations of CXC chemokines (i.e., CXCL8 and CXCL12) were differentially affected. Thus, although plasma CXCL8 concentrations were not affected by “history of AUD” (Figure 1A), we observed a main effect of this factor on CXCL12 concentrations [*F*_(1,129)_ = 11.589, *p* < 0.001] (Figure 1B). Consequently, a significant reduction in plasma CXCL12 concentrations was detected in the alcohol group compared to the control group [285.76 (95% CI = 266.07–307.61) pg/mL and 349.95 (95% CI = 319.15–384.59) pg/mL, respectively].

CX_3_CL1 had an expression profile similar to that observed for CXCL12. Therefore, plasma CX_3_CL1 concentrations were also significantly affected by “history of AUD” [*F*_(1,129)_ = 6.124, *p* = 0.015] (Figure 1C) and a significant decrease in the plasma concentrations was also observed in the alcohol group compared to the control group [4.592 (95% CI = 4.140–5.105) pg/mL and 5.689 (95% CI = 4.966–6.516) pg/mL, respectively].

Regarding the CC chemokine expression (i.e., CCL2, CCL3, and CCL11), plasma concentrations were not affected by “history of AUD” as observed in the Figures 1D–F. However, the statistical analysis revealed a main effect of “sex” on CCL3 concentrations [*F*_(1,129)_ = 6.679, *p* = 0.011] (Figure 1G) and a significant interaction between “history of AUD” and “sex” on CCL11 concentrations [*F*_(1,129)_ = 4.922, *p* = 0.028] (Figure 1H). Plasma CCL3 concentrations were higher in women than men with no distinction between both alcohol and control groups. In the case of CCL11, paired comparisons revealed that CCL11 concentrations were decreased in female compared to male subjects but only in the alcohol group (*p* < 0.001). Furthermore, the “age” was significantly related to CCL11 concentrations [*F*_(1,129)_ = 14.969, *p* < 0.001] and correlation analysis indicated a positive association between “age” and CCL11 concentrations (*r* = +0.299, *p* < 0.001) in the total sample. Additional correlation analyses between “age” and CCL11 concentrations were conducted in different groups according to each factor (“history of AUD” or “sex”) and we observed an enhanced association in the control group (*r* = +0.423, *p* = 0.002) and in male participants (*r* = +0.384, *p* = 0.001).

### Alcohol-Related Variables and Plasma Chemokine Concentrations in Abstinent AUD Patients

We evaluated the relative effects of variables associated with AUD and their interactions on chemokine concentrations in the alcohol group using a two-way ANCOVA. The independent variables that we included were as follows: alcohol-induced “liver and pancreas diseases” (*N* = 20 disease and *N* = 65 no disease), “sex” (*N* = 58 men and *N* = 27 women), “AUD symptom severity,” length of “abstinence,” and “age.” For all analyses, “psychiatric medication” was included as confounding variable. All variables were examined to ensure that statistical assumptions were met.

“AUD symptom severity,” length of “abstinence,” and “age” were fixed at the following mean values: 6.9 (SD = 2.3) criteria for AUD, 9.2 (SD = 11.8) months of abstinence, and 47.2 (SD = 7.3) years. Plasma concentrations of CXCL12, CX_3_CL1, CCL3, and CCL2 were not affected by any of the variables that were evaluated in abstinent AUD patients. However, we found significant effects on CXCL8 and CCL11 concentrations.

#### Liver and Pancreas Diseases and CXCL8 Concentrations in Abstinent AUD Patients

**Figure 2:**
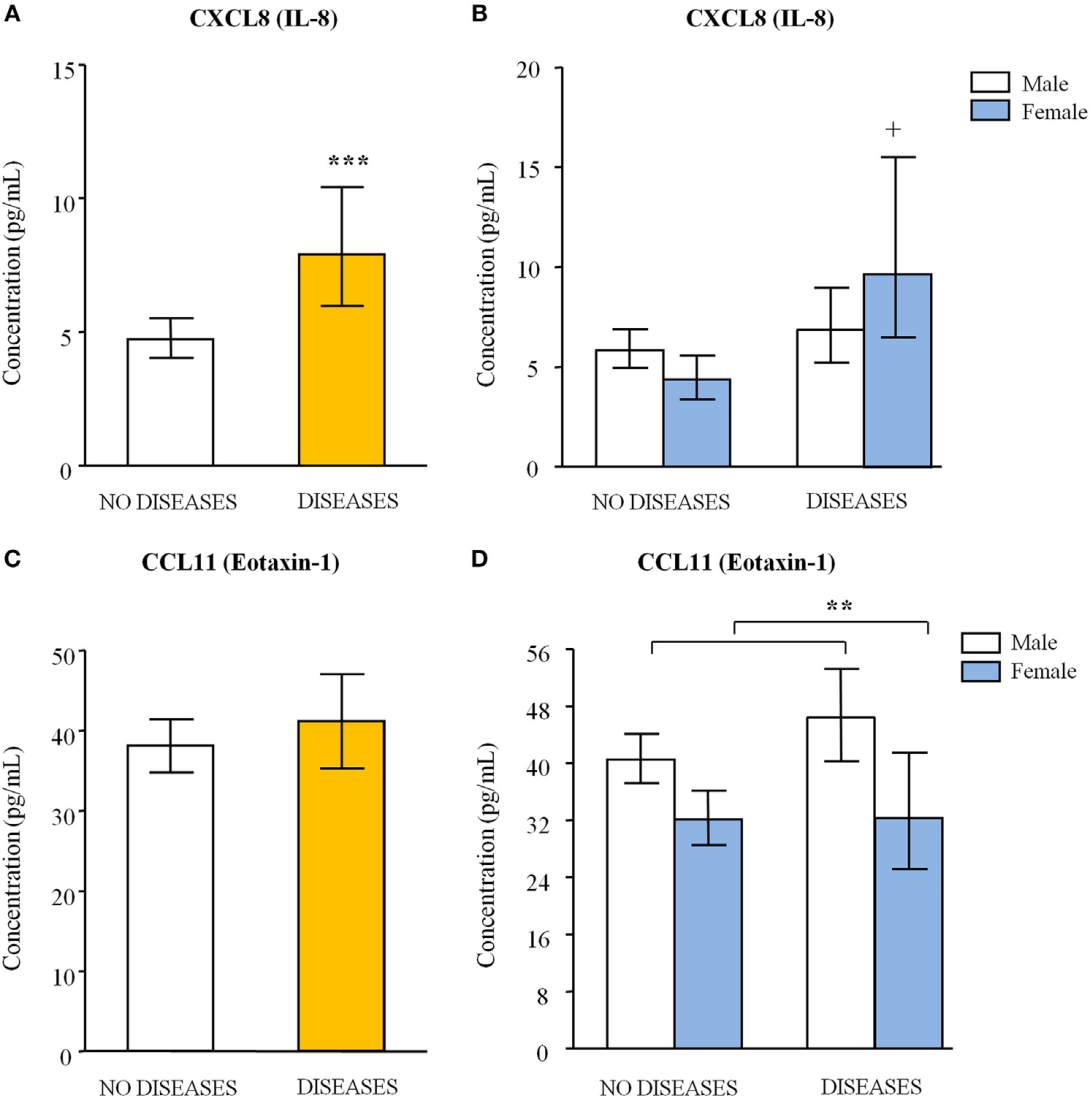
**Plasma concentrations of CXCL8 [interleukin-8 (IL-8)] and CCL11 (eotaxin-1) in abstinent alcohol use disorders patients with liver and/or pancreas diseases**. **(A)** CXCL8 (IL-8) concentrations according to “liver and pancreas diseases”; **(B)** CXCL8 (IL-8) concentrations according to “liver and pancreas diseases” and “sex”; **(C)** CCL11 (eotaxin-1) concentrations according to “liver and pancreas diseases”; and **(D)** CCL11 (eotaxin-1) concentrations according to “liver and pancreas diseases” and “sex.” Bars are estimated marginal means and 95% CI (picograms per milliliter). Data were analyzed by two-way analysis of covariance and **p < 0.01 and ***p < 0.001 denote significant main effect of “sex” and “liver and pancreas diseases,” respectively. ^+^*p* < 0.05 denotes significant differences compared to female patients with no diseases because there was an interaction of factors.

CXCL8 concentrations were significantly affected by “liver and pancreas diseases” [*F*_(1,77)_ = 11.409, *p* < 0.001] (Figure 2A) but there was a disordinal interaction between “liver and pancreas diseases” and “sex” [*F*_(1,77)_ = 5.773, *p* = 0.019] (Figure 2B). Thus, multiple paired comparisons revealed a significant increase in CXCL8 concentrations in women diagnosed with alcohol-induced diseases (*N* = 5) compared to female patients with no diseases (*N* = 22) (*p* < 0.05). In addition, “psychiatric medication” was significantly related to CXCL8 concentrations [*F*_(1,77)_ = 6.500, *p* = 0.013] and patients treated with medication (*N* = 13 with alcohol-induced diseases and *N* = 47 with no diseases) displayed higher CXCL8 concentrations relative to patients with no medication (*N* = 7 with alcohol-induced diseases and *N* = 17 with no diseases) during the last year.

Because alcoholic liver and pancreas damage are characterized by changes in certain circulating markers we examined AST/GOT, ALT/GPT, GGT, pancreatic α-amylase, and pancreatic lipase levels in patients and their degree of association with chemokine concentrations. We found an overall increase of plasma levels of these markers in AUD patients diagnosed with cirrhosis, hepatitis, and/or pancreatitis compared to AUD patients with no diseases, whereas control subjects had lower levels of these circulating markers than AUD patients (Table 2). Regarding correlation studies, only CXCL8 concentrations were significantly correlated to AST/GOT (*r* = +0.456, *p* < 0.001) and GGT (*r* = +0.647, *p* < 0.001) after multiple testing correction with Bonferroni. Although the subclinical affectation of the liver and pancreas in AUD patients could induce changes in the expression of other chemokines, we observed no associations in the total sample (control and alcohol group) with the exception of CXCL8 with AST/GOT (*r* = +0.394, *p* = 0.001) and CXCL8 and GGT (*r* = +0.563, *p* < 0.001).

**Table 2:** **Estimated marginal means of circulating markers related to liver and pancreas functions in abstinent alcohol use disorders patients and controls**.

Variable		Alcohol with diseases (*N* = 20)	Alcohol with no diseases (*N* = 65)	Control (*N* = 55)	*p-*Value^a^
Aspartate transaminase [mean (SD)]	Reference ranges [0.0–40.0] IU/L	46.11 (4.01)^+^	29.49 (2.24)	27.94 (3.14)	**0.001**
Alanine transaminase [mean (SD)]	Reference ranges [0.0–40.0] IU/L	49.16 (4.78)^++^	32.34 (2.67)	30.00 (3.74)	**0.004**
Gamma-glutamyltransferase [mean (SD)]	Reference ranges [0.0–45.0] IU/L	47.84 (7.18)^++^	37.18 (4.00)^+++^	19.48 (5.62)	**0.005**
Pancreatic α-amylase [mean (SD)]	Reference ranges [5.0–100.0] IU/L	77.00 (9.16)	69.33 (5.11)	59.84 (7.17)	0.316
Pancreatic lipase [mean (SD)]	Reference ranges [0.0–67.0] IU/L	35.42 (3.53)	39.49 (1.97)^+++^	29.09 (2.76)	**0.011**

*Underlined numbers denote out of reference ranges (normal ranges)*.

*Bold numbers denote significant differences (*p* < 0.05)*.

*^a^p-Value from one-way analysis of variance using F statistic*.

*^+,++,+++^Significant differences compared to the control group after a multiple post hoc Dunnett/Tukey test*.

#### Sex Differences and CCL11 Concentrations in Abstinent AUD Patients

Regarding CCL11, despite there was no main effect of “liver and pancreas diseases” (Figure 2C), concentrations were strongly affected by “sex” [*F*_(1,77)_ = 8.298, *p* = 0.005] (Figure 2D) with no interaction. Estimated means indicated that male abstinent patients displayed higher CCL11 concentrations than female patients [43.35 (95% CI = 39.90–46.99) pg/mL and 32.21 (95% CI = 27.93–37.07) pg/mL, respectively]. Furthermore, we detected a significant effect of “abstinence” [*F*_(1,77)_ = 4.239, *p* = 0.041] and we found a negative correlation between CCL11 concentrations and length of abstinence but this association failed to reach statistical significance. Similar to previous analysis of CCL11 concentrations in the total sample, the “age” was also significantly related to CCL11 concentrations [*F*_(1,77)_ = 8.891, *p* = 0.004].

### Psychiatric Comorbidity in Abstinent AUD Patients

Since chemokines have been linked to psychiatric disorders, we studied the high prevalence of psychiatric comorbidity (76%) diagnosed in abstinent AUD patients. Thus, the alcohol group was divided into comorbid and non-comorbid patients according to lifetime diagnosis of substance use disorders (45%) and mental disorders (72%) for characterization (Table 3). Additionally, the presence of psychiatric comorbidity was also studied in women and men considering the potential impact of sex differences on psychiatric disorders (Table 4).

**Table 3:** **Baseline sociodemographic variables and psychiatric disorders in abstinent alcohol use disorders patients grouped according to psychiatric comorbidity**.

Variable		Alcohol (*N* = 85)					
		Comorbid substance use disorders			Other comorbid mental disorders		
		No (*N* = 47)	Yes (*N* = 38)	*p*-Value	No (*N* = 24)	Yes (*N* = 61)	*p*-Value
Age [mean (SD)]	Years	48.9 (6.1)	45.1 (8.1)	**0.017^a^**	47.8 (6.6)	46.9 (7.6)	0.621^a^

BMI [mean (SD)]	kg/m^2^	25.8 (3.8)	25.58 (3.9)	0.823^a^	25.6 (3.6)	25.7 (4.00)	0.835^a^

Sex [*N* (%)]	Women	20 (42.6)	7 (18.4)	**0.021^b^**	6 (0.25)	21 (0.35)	0.450^b^
	Men	27 (57.4)	31 (81.6)		18 (0.75)	40 (0.65)	

Liver and pancreas diseases [*N* (%)]	Steatosis	8 (17.0)	1 (2.6)	**0.024^b^**	5 (20.8)	4 (6.6)	**0.017^b^**
	Cirrhosis	5 (10.6)	1 (2.6)		4 (16.7)	2 (3.3)	
	Pancreatitis	4 (8.5)	1 (2.6)		2 (8.3)	3 (4.9)	

Lifetime alcohol use disorders [*N* (%)]	Abuse	1 (2.1)	4 (10.5)	0.109^b^	0 (0.0)	5 (8.2)	0.220^b^
	Dependence	5 (10.6)	1 (2.6)		1 (4.2)	6 (9.8)	
	Both	41 (87.2)	33 (86.8)		23 (95.8)	50 (82.0)	

Smoking [*N* (%)]	No	5 (10.6)	5 (13.2)	0.934^b^	1 (4.2)	8 (13.1)	0.469^b^
	Yes	34 (72.3)	27 (71.1)		20 (83.3)	45 (73.8)	
	Former smoker	8 (17.0)	6 (15.8)		3 (12.5)	8 (13.1)	

Other lifetime substance use disorders [*N* (%)]	No	47 (100.0)	0 (0.0)	–	20 (83.3)	27 (44.3)	**0.001^b^**
	Yes	0 (0.0)	38 (100.0)		4 (16.7)	34 (55.7)	
	Cocaine	0 (0.0)	28 (73.7)	–	3 (12.5)	25 (41.0)	**0.020^b^**
	Cannabis	0 (0.0)	15 (39.5)	–	2 (8.3)	13 (21.3)	0.214^b^
	Heroin	0 (0.0)	3 (7.9)	–	0 (0.0)	3 (4.9)	0.555^b^
	Sedatives	0 (0.0)	2 (5.3)	–	0 (0.0)	2 (3.3)	1.000^b^
	Others	0 (0.0)	7 (18.4)	–	2 (8.3)	5 (8.2)	1.000^b^

Common lifetime mental disorders [*N* (%)]	No	20 (42.6)	4 (10.5)	**<0.001^b^**	24 (100.0)	0 (0.0)	–
	Yes	27 (57.4)	34 (89.5)		0 (0.0)	61 (100.0)	
	Mood	20 (42.5)	22 (57.9)	0.194^b^	0 (0.0)	42 (68.9)	–
	Anxiety	7 (14.9)	9 (23.7)	0.404^b^	0 (0.0)	16 (26.2)	–
	Psychosis	4 (8.6)	2 (5.3)	0.687^b^	0 (0.0)	6 (9.8)	–
	Personality	5 (10.6)	16 (42.1)	**0.001^b^**	0 (0.0)	21 (34.4)	–
	ADHD (childhood)	4 (8.5)	18 (47.4)	**<0.001^b^**	0 (0.0)	22 (36.1)	–

*BMI, body mass index; ADHD, attention deficit hyperactivity disorder*.

*Bold numbers denote significant differences (*p* < 0.05)*.

*^a^p-Value from Student’s t-test. Age: t = 2.47, df = 83*.

*^b^p-Value from Fisher’s exact test or chi-square test*.

**Table 4:** **Baseline sociodemographic variables and psychiatric disorders in abstinent alcohol use disorders patients diagnosed with psychiatric comorbidity grouped according to sex**.

Variable		Alcohol (*N* = 85)					
		Comorbid substance use disorders			Other comorbid mental disorders		
		Male (*N* = 31)	Female (*N* = 7)	*p*-Value	Male (*N* = 40)	Female (*N* = 21)	*p*-Value
Age [mean (SD)]	Years	44.7 (8.3)	46.7 (7.7)	0.562^a^	45.9 (8.3)	48.9 (5.6)	0.148^a^

BMI [mean (SD)]	kg/m^2^	26.1 (4.0)	23.3 (3.3)	0.090^a^	26.6 (4.0)	24.0 (3.5)	**0.013^a^**

Liver and pancreas diseases [*N* (%)]	No	28 (90.3)	7 (100.0)	1.000^b^	35 (87.5)	17 (81.0)	0.706^b^
	Yes	3 (9.7)	0 (0.0)		5 (12.5)	4 (19.0)	
	Steatosis	1 (3.2)	0 (0.0)	–	2 (5.0)	2 (9.5)	–
	Cirrhosis	1 (3.2)	0 (0.0)	–	1 (2.5)	1 (4.8)	–
	Pancreatitis	1 (3.2)	0 (0.0)	–	2 (5.0)	1 (4.8)	–

Lifetime alcohol use disorders [*N* (%)]	Abuse	3 (9.7)	1 (14.3)	1.000^b^	3 (7.5)	2 (9.5)	1.000^b^
	Dependence	28 (90.3)	6 (85.7)		37 (92.5)	19 (90.5)	

Smoking [*N* (%)]	No	4 (12.9)	1 (14.3)	0.568^b^	4 (10.0)	4 (19.0)	0.574^b^
	Yes	23 (74.2)	4 (57.1)		31 (77.5)	14 (66.7)	
	Former smoker	4 (12.9)	2 (28.6)		5 (12.5)	3 (14.3)	

Other lifetime substance use disorders [*N* (%)]	No	0 (0.0)	0 (0.0)	–	13 (32.5)	14 (66.7)	**0.015^b^**
	Yes	31 (100.0)	7 (100.0)		27 (67.5)	7 (33.3)	
	Cocaine	25 (80.6)	3 (42.9)	0.063^b^	22 (55.0)	3 (14.3)	**0.003^b^**
	Cannabis	13 (41.9)	2 (28.6)	0.681^b^	11 (27.5)	2 (9.5)	0.187^b^
	Heroin	1 (3.2)	2 (28.6)	0.081^b^	1 (2.5)	2 (9.5)	0.270^b^
	Sedatives	1 (3.2)	1 (14.3)	0.339^b^	1 (2.5)	1 (4.8)	1.000^b^
	Others	7 (3.2)	0 (0.0)	0.309^b^	5 (12.5)	0 (0.0)	0.154^b^

Common lifetime mental disorders [*N* (%)]	No	4 (12.9)	0 (0.0)	1.000^b^	0 (0.0)	0 (0.0)	–
	Yes	27 (87.1)	7 (100.0)		40 (100.0)	21 (100.0)	
	Mood	16 (51.6)	6 (85.7)	0.203^b^	26 (65.0)	16 (76.2)	0.561^b^
	Anxiety	8 (25.8)	1 (14.3)	1.000^b^	11 (27.5)	5 (23.8)	1.000^b^
	Psychosis	2 (6.5)	0 (0.0)	1.000^b^	4 (10.0)	2 (9.5)	1.000^b^
	Personality	11 (35.5)	5 (71.4)	0.108^b^	14 (35.0)	7 (33.3)	1.000^b^
	ADHD (childhood)	17 (54.8)	1 (14.3)	0.093^b^	19 (47.5)	3 (14.3)	**0.012^b^**

*BMI, body mass index; ADHD, attention deficit hyperactivity disorder*.

*Bold numbers denote significant differences (*p* < 0.05)*.

*^a^p-Value from Student’s t-test*.

*^b^p-Value from Fisher’s exact test or chi-square test*.

#### Prevalence of Comorbid Substance Use Disorders in Abstinent AUD Patients

As is shown in Table 3, abstinent AUD patients diagnosed with comorbid substance use disorders [predominantly cocaine (74%) and cannabis (40%)] were younger men with low incidence of liver and pancreas diseases relative to patients with no additional substance use disorders. Interestingly, patients with comorbid substance use disorders had a higher prevalence of common mental disorders (90%), mainly personality disorders (42%) and ADHD (47%), than non-comorbid patients (57%). When patients with comorbid substance use disorders were grouped according to sex (*N* = 31 men and *N* = 7 women) (Table 4), we observed no differences relative to sex but there was a trend for female patients to use other substances of abuse with no preference and to display high prevalence of mood (86%) and personality (71%) disorders.

#### Prevalence of Comorbid Mental Disorders in Abstinent AUD Patients

Regarding patients diagnosed with other comorbid mental disorders [mainly mood disorders (69%)], we found no differences in age and sex compared to patients with no disorders (Table 3). However, there was a low incidence of liver and pancreas diseases in comorbid patients. In this case, patients with common mental disorders displayed a higher prevalence of other substance use disorders (56%) than patients with no mental disorders (17%), predominately cocaine use disorders (41%). According to sex, patients diagnosed with comorbid mental disorders were balanced between women and men and some differences were found (Table 4). Thus, female patients with mental disorders had a decreased prevalence of substance use disorders (33%) compared to male patients (68%). Although we observed a similar diagnosis of mental disorders in women and men lower, there was a low prevalence of ADHD in female patients (14%).

Finally, a more precise diagnosis of common mental disorders according to sex is shown in Table 5, distinguishing between primary and alcohol-induced disorders for mood, anxiety, and psychotic disorders.

**Table 5:** **Prevalence of mental disorders in abstinent alcohol use disorders patients with comorbid mental disorders grouped according to sex**.

Mental disorders		Alcohol subgroup with comorbid mental disorders		
		Male (*N* = 40)	Female (*N* = 21)	Total (*N* = 61)
Mood disorders	No	14 (35.0)	5 (23.8)	19 (31.1)
	Yes	26 (65.0)	16 (76.2)	42 (68.9)
	Primary	10	9	19
	Alcohol-induced	17	11	28
	Both	1	4	5
	Major depression	26	16	42
	Dysthymia	0	1	1
	Mania	3	0	3
	Hipomania	1	0	1
	Cyclothymia	0	0	0

Anxiety disorders	No	30 (75.0)	16 (76.2)	46 (75.4)
	Yes	10 (25.0)	5 (23.8)	15 (24.6)
	Primary	6	5	11
	Alcohol-induced	5	0	5
	Both	1	0	1
	Specific phobia	0	0	0
	Social phobia	1	0	1
	Panic disorder/agoraphobia	6	2	8
	Generalized anxiety disorder	2	1	3
	Obsessive–compulsive disorder	0	0	0
	Posttraumatic stress disorder	2	2	4

Psychotic disorders	No	36 (90.0)	19 (90.5)	55 (90.2)
	Yes	4 (10.0)	2 (9.5)	6 (9.8)
	Primary	1	1	2
	Alcohol-induced	3	1	4
	Both	0	0	0
	Schizoaffective disorder	0	0	0
	Brief psychotic disorder	1	0	1
	Delusional disorder	0	0	0
	Mood disorder with psychotic feature	3	2	5
	Psychosis due to a medical condition	0	0	0
	Psychosis not otherwise specified	1	0	1

Eating disorders	No	40 (100.0)	19 (90.5)	59 (96.7)
	Yes	0 (0.0)	2 (9.5)	2 (3.3)
	Anorexia nervosa	0	0	0
	Bulimia nervosa	0	2	2
	Binge eating disorder	0	0	0

Personality disorders	No	26 (65.0)	14 (52.4)	40 (66.7)
	Yes	14 (35.0)	7 (47.6)	21 (33.3)
	Borderline disorder	8	7	15
	Antisocial disorder	7	0	7

ADHD	No	21 (52.5)	18 (85.7)	39 (63.9)
	Yes	19 (47.5)	3 (14.3)	22 (36.1)

*ADHD, attention deficit hyperactivity disorder*.

### Psychiatric Comorbidity and Plasma Chemokine Concentrations

The impact of psychiatric comorbidity on chemokine expression was analyzed using a two-way ANCOVA with the following factors: the diagnosis of “comorbid substance use disorders” or diagnosis of “comorbid mental disorders” and “sex.” These independent variables were controlled for “age.” For all analyses, “psychiatric medication” was also included as confounding variable. All variables were examined to ensure that statistical assumptions were met.

#### Comorbid Substance Use Disorders and Plasma Chemokine Concentrations

**Figure 3:**
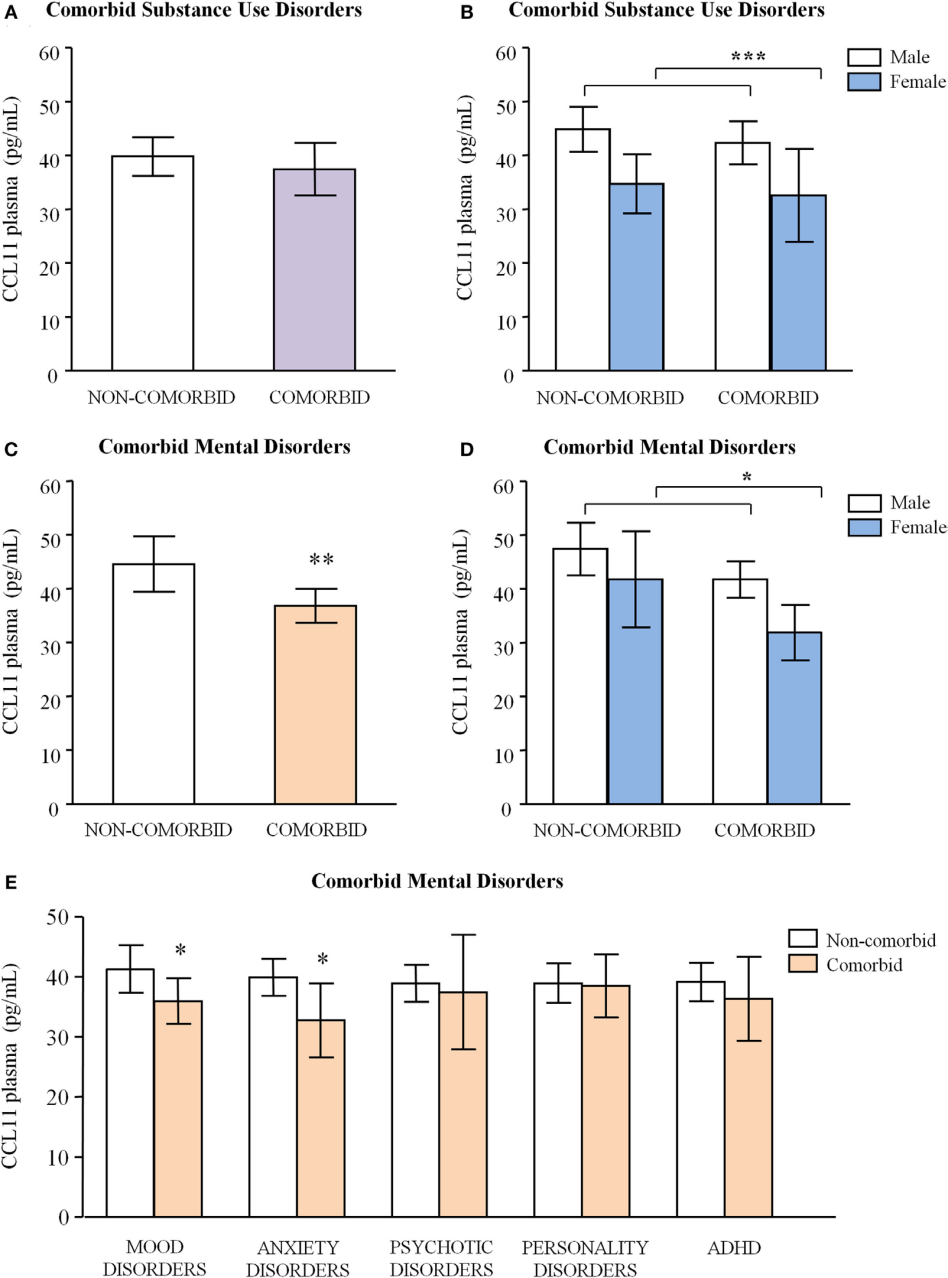
**Plasma concentrations of CCL11 (eotaxin-1) in abstinent alcohol use disorders patients according to psychiatric comorbidity**. **(A)** CCL11 (eotaxin-1) concentrations according to “comorbid substance use disorders”; **(B)** CCL11 (eotaxin-1) concentrations according to “comorbid substance use disorders” and “sex”; **(C)** CCL11 (eotaxin-1) concentrations according to “comorbid mental disorders”; **(D)** CCL11 (eotaxin-1) concentrations according to “comorbid mental disorders” and “sex”; and **(E)** CCL11 (eotaxin-1) concentrations according to mood disorders, anxiety, psychotic disorders, personality disorders, and ADHD. Bars are estimated marginal means and 95% CI (picograms per milliliter). Data were analyzed by two-way analysis of covariance and *p < 0.05, **p < 0.01, and ***p < 0.001 denote significant main effect of factors.

Plasma concentrations of chemokines were not found to be affected by any of the variables evaluated, with the exception of CCL11. We observed no main effect of “comorbid substance use disorders” on CCL11 concentrations (Figure 3A) but there was a main effect of “sex” [*F*_(1,79)_ = 11.132, *p* < 0.001] with no interaction between both factors (Figure 3B). As expected, “age” was also significantly related to CCL11 concentrations [*F*_(1,79)_ = 5.572, *p* = 0.021].

#### Comorbid Mental Disorders and Plasma Chemokine Concentrations

Regarding the diagnosis of comorbid mental disorders, we observed significant effects on CCL11 concentrations. Thus, statistical analysis revealed a significant main effect of “comorbid mental disorders” [*F*_(1,79)_ = 7.559, *p* = 0.007] on CCL11 concentrations as shown in Figure 3C, and patients diagnosed with comorbid mental disorders had decreased CCL11 concentrations relative to non-comorbid patients [36.79 (95% CI = 33.59–39.99) pg/mL and 44.58 (95% CI = 39.46–49.70) pg/mL, respectively]. Again, there was a significant effect of “sex” [*F*_(1,79)_ = 6.524, *p* = 0.013] on CCL11 concentrations (Figure 3D) and the covariate “age” was also related to CCL11 concentrations [*F*_(1,79)_ = 6.752, *p* = 0.011].

### Comorbid Mental Disorders and Plasma CCL11 Concentrations in Abstinent AUD Patients

As a continuation of the effects of comorbid mental disorders on CCL11 expression, we evaluated the contribution of the most prevalent mental disorders.

We analyzed the effects of these psychiatric disorders on CCL11 including “sex” and controlling for “age” and “psychiatric medication” (Figure 3E). Thus, a two-way ANCOVA revealed that CCL11 concentrations were significantly affected by “mood disorders” [*F*_(1,79)_ = 4.627, *p* = 0.034] and the estimated CCL11 mean was lower in patients diagnosed with mood disorders than in patients with no mood disorders [35.92 (95% CI = 32.15–39.68) pg/mL and 41.28 (95% CI = 37.34–45.23) pg/mL, respectively].

Plasma CCL11 concentrations were also affected by “anxiety” [*F*_(1,79)_ = 4.941, *p* = 0.028] and the estimated marginal mean of CCL11 was lower in patients diagnosed with lifetime anxiety than in patients with no anxiety [32.71 (95% CI = 26.55–38.88) pg/mL and 39.86 (95% CI = 36.19–42.93) pg/mL, respectively].

Not surprisingly, the statistical analysis of the effects of these common mental disorders on CCL11 expression revealed a significant main effect of “sex” with no interaction. Therefore, female patients displayed lower concentrations than male patients (for clarity, statistics related to “sex” were not included to prevent repetitive outputs). Therefore, female abstinent patients diagnosed with lifetime anxiety (*N* = 5) and/or displayed the lowest concentrations of CCL11 [26.38 (95% CI = 16.47–36.29) pg/mL].

### Plasma Chemokine Concentrations in Rats Exposed to Ethanol

CXCL11, CX_3_CL1, and CCL12 concentrations were examined in Wistar rats exposed to repeated and acute ethanol. In addition to ethanol, CCL11 concentrations were also examined in abstinent rats exposed to acute stress induced by immobilization. To simplify the experiments, we included only male rats at the same age.

#### Plasma Chemokine Concentrations in Rats Exposed to Repeated Ethanol

**Figure 4:**
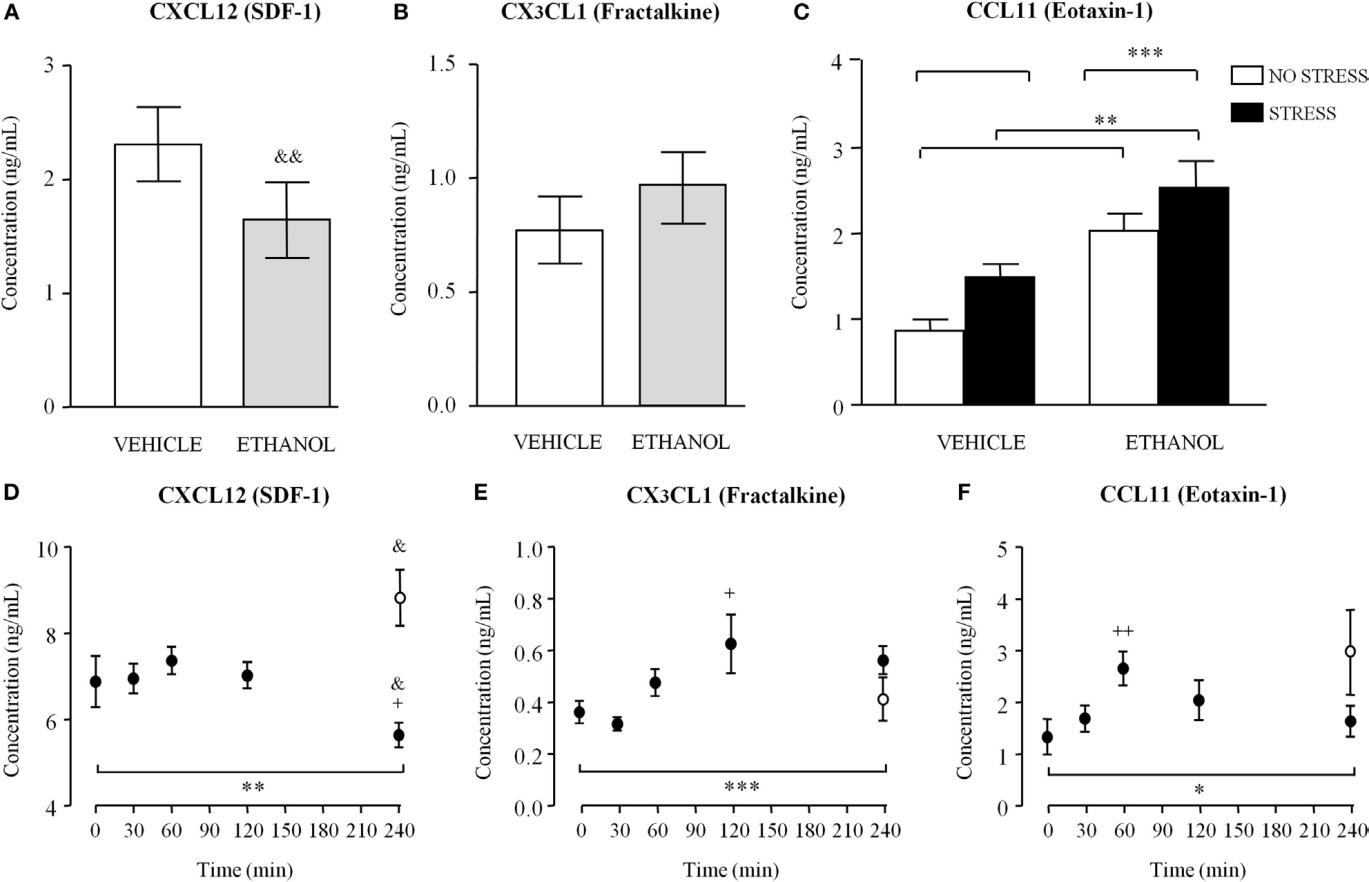
**Plasma concentrations of CXCL12 [stromal cell-derived factor-1 (SDF-1)], CX_3_CL1 (fractalkine), and CCL11 (eotaxin-1) in male Wistar rats exposed to ethanol and acute stress**. **(A)** CXCL12 (SDF-1) and **(B)** CX_3_CL1 (fractalkine) concentrations were determined in rats exposed to ethanol (3 g/kg, i.g.) during 4 weeks or vehicle. **(C)** CCL11 (eotaxin-1) concentrations were determined in rats exposed to ethanol (3 g/kg, i.g.) during 4 weeks or vehicle with/without acute stress before ethanol exposure. Bars are means and SEM (nanograms per milliliter). CXCL12 and CX_3_CL1 concentrations were analyzed by Student’s *t*-test and ^&&^*p* < 0.01 denotes significant differences compared to the vehicle group. CCL11 concentrations were analyzed by two-way analysis of variance (ANOVA) and **p < 0.01 and ***p < 0.001 denote significant main effect of “stress” and “ethanol exposure,” respectively. **(D)** CXCL12 (SDF-1); **(E)** CX_3_CL1 (fractalkine); and **(F)** CCL11 (eotaxin-1) concentrations were determined in rats exposed to acute ethanol (3 g/kg, i.g.) at 0, 30, 60, 120, and 240 min after ethanol exposure. Circles are means and SEM (nanograms per milliliter). CXCL12, CX_3_CL1, and CCL11 concentrations were analyzed by one-way ANOVA and *p < 0.05, **p < 0.01, and ***p < 0.001 denote significant main effect of “time.” ^+^*p* < 0.05 and ^++^*p* < 0.01 denote significant differences compared to *t* = 0 min. White circles are means and SEM (nanograms per milliliter) at *t* = 240 min with no ethanol exposure and concentrations were analyzed by Student’s *t*-test. ^&^*p* < 0.05 denotes significant differences compared to *t* = 0 or 240 min with no ethanol.

Because only CXCL12 and CX_3_CL1 concentrations were found to be altered in the plasma of abstinent AUD patients relative to the control group in humans, we tested whether these chemokines were also affected by ethanol administration in abstinent Wistar rats. Two weeks after the last ethanol administration alcohol-exposed rats had lower CXCL12 concentrations than vehicle-treated rats, resulting in a significant decrease [*t*_(38)_ = 2.99, *p* = 0.005] (Figure 4A). However, we observed no significant differences in plasma CX_3_CL1 concentrations between both groups (Figure 4B).

In humans, we found that the CCL11 expression was significantly affected by lifetime mental disorders, especially mood disorders and anxiety disorders. In order to investigate the potential link of this chemokine to emotional behaviors, we investigated whether these effects on CCL11 concentrations were also observed in male Wistar rats exposed to acute stress induced by immobilization and treated with ethanol. As shown in Figure 4C, a two-way ANOVA revealed a main effect of “ethanol exposure” [*F*_(1,73)_ = 27.02, *p* < 0.001] with higher CCL11 concentrations in alcohol-exposed rats compared to vehicle rats and a main effect of early “stress” [*F*_(1,73)_ = 6.975, *p* < 0.01] with higher CCL11 concentrations in rats exposed to stress compared to rats without restraint. There was no interaction between both factors.

#### Plasma Chemokine Concentrations in Rats Exposed to Acute Ethanol

To study the impact of acute ethanol on these chemokines, another set of male Wistar rats were treated with ethanol (i.g.) and CXCL12, CX_3_CL1, and CCL11 concentrations were determined in the plasma during 4 h at different times (0, 30, 60, 120, and 240 min). Data were analyzed using one-way ANOVA with *post hoc* comparisons.

As shown in Figure 4D, there was a significant main effect of “time” on CXCL12 concentrations across the experimental groups [*F*_(4,67)_ = 3.871, *p* = 0.007] but the *post hoc* tests only found a significant decrease in CXCL12 at 240 min after receiving ethanol compared to the *t* = 0 min (*p* < 0.05). An additional group of rats with no ethanol exposure was examined at 240 min after beginning the experiment and this group displayed higher CXCL12 concentrations than the *t* = 0 min [*t*_(20)_ = 2.22, *p* = 0.038]. Consequently, ethanol-treated rats showed lower CXCL12 concentrations relative to naive rats at 240 min [*t*_(20)_ = 5.17, *p* < 0.001].

Regarding CX_3_CL1, one-way ANOVA revealed a significant main effect of “time” in the subgroups [*F*_(4,65)_ = 4.166, *p* < 0.001] (Figure 4E). In fact, CX_3_CL1 concentrations were significantly increased at 120 min after ethanol exposure compared to the *t* = 0 min (*p* < 0.05). In this case, we found no differences in the CX_3_CL1 expression when naïve groups at 0 and 240 min were compared. Furthermore, ethanol-treated rats and naïve rats did not differ in CX_3_CL1 concentrations at 240 min.

Similar to previous analyses, there were significant differences in the CCL11 expression among groups according to “time” after ethanol exposure [*F*_(4,65)_ = 2.950, *p* = 0.027] (Figure 4F). The *post hoc* tests indicated a significant increase in CCL11 concentrations at 60 min after receiving ethanol relative to rats at *t* = 0 min (*p* < 0.01). Regarding the naïve groups, rats with no ethanol exposure showed no differences in CCL11 at *t* = 0 and 240 min. There were also no differences in CCL11 concentrations when ethanol-treated and naïve rats were compared at 240 min.

## Discussion

While the association of immune signaling and psychiatric disorders is widely investigated, its association with addiction is still a field under exploration. We lack essential information concerning how immune signaling in the brain contributes to the neuroadaptations imposed by chronic substance use. This is especially relevant in the case of alcohol that can directly activate neuroimmune processes (26). In the present study, we measured plasma chemokines in a well-phenotyped cohort of subjects with AUD in abstinence treated in an outpatient setting. This cohort was characterized for the high presence of psychiatric comorbidity although we observed sex differences in the prevalence of comorbid substance use disorders and mental disorders. The main findings are as follows: (i) abstinent AUD patients had lower concentrations of CXCL12 and CX_3_CL1 compared to control subjects whereas CCL11 concentrations were strongly decreased in female AUD patients; (ii) CXCL8 concentrations were elevated in patients with cirrhosis, steatosis, or pancreatitis, particularly in female AUD patients, and there was a positive correlation to AST/GOT and GGT; (iii) CCL11 concentrations were found to be decreased in abstinent AUD patients with comorbid mental disorders (i.e., mood and anxiety disorders); (iv) there was a strong and permanent effect of sex and age on plasma CCL11 concentrations in the alcohol group throughout the present study; (v) studies conducted in male rats exposed to repeated ethanol revealed similar changes in plasma CXCL12 concentrations than those observed in humans during abstinence during abstinence and acute ethanol exposure also induced a decrease in CXCL12; and (vi) rats exposed to repeated ethanol and early stress (restraint) displayed changes in CCL11 concentrations, although the changes were opposite to those observed in humans.

### Abstinent AUD Patients Have Altered CXCL12/SDF-1 and CX_3_CL1/Fractalkine Concentrations in the Plasma

Patients with a history of AUD displayed lower plasma concentrations of CXCL12 and CX_3_CL1 relative to control subjects with no pathological use of substances or substance use disorders. Various studies have reported an association between both chemokines and abused drugs, mainly with cocaine. Recently, our group has reported a decrease in the plasma CXCL12 concentrations and a positive correlation between CX_3_CL1 concentrations and the cocaine symptom severity in abstinent cocaine-addicted subjects from outpatient setting (6, 21). In agreement with these clinical data, changes in the plasma concentrations of both chemokines have been observed in mice exposed to cocaine (21). However, another study in chronic cocaine users showed increased CXCL12 concentrations in the moment of admission for treatment, although they were decreased after 1 month of cocaine withdrawal (27).

Unlike cocaine, there are scarce exploratory studies examining the circulating concentrations of CXCL12 after alcohol exposure. In fact, these studies conducted in humans (28) and mice (29) have reported high circulating concentrations of CXCL12 after moderate alcohol consumption. By contrast, here we found low concentrations of CXCL12 in AUD patients but also in rats exposed to repeated ethanol after 1 week of abstinence. Moreover, rats treated with acute ethanol displayed a decrease in CXCL12 levels after 4 h, which is congruent with the data from repeated experiments. Regarding plasma CX_3_CL1, most studies on the effects of alcohol are focused on the CNS (30, 31) and pancreas (32, 33) but a recent study has reported elevated concentrations of CX_3_CL1 in the serum of mice treated chronically (5 months) with ethanol (26), which is in opposition to our data in AUD patients but compatible with our preclinical data since ethanol-treated rats had higher plasma CX_3_CL1 concentrations, especially in rats acutely treated. However, it is important to indicate that species, doses and intensity of the exposure were not comparable in between these studies and that further research is needed to understand the time-course of the changes in chemokines in plasma after acute and chronic alcohol exposure.

Therefore, although increased circulating levels of CXCL12 and CX_3_CL1 after alcohol exposure are opposed to the present data, there are marked differences regarding the time of the last alcohol exposure (abstinence) and the AUD severity in the clinical studies. Our study has been performed in patients diagnosed with AUD in a prolonged abstinence (about 9 months) and a history of heavy alcohol consumption, whereas the above cited studies in humans were performed in high cardiovascular risk patients reporting moderate alcohol consumption. This discrepancy might indicate that both chemokines fluctuate regarding the duration of abstinence and intensity of alcohol use.

In addition to CXCL12 and CX_3_CL1, differences in CCL11 concentrations were found between men and women but only in the alcohol group, which indicated the presence of a sexual dimorphism in the circulating CCL11 concentrations in response to a history of pathological alcohol exposure and/or AUD. By contrast, we observed differences in the expression of this chemokine in male rats after ethanol exposure. Thus, we found increased levels of CCL11 in rats treated with acute and repeated ethanol compared to control rats.

### Age and Sex Differences Influence CCL3/MIP-1α and CCL11/Eotaxin-1 Concentrations in the Plasma

Age and sex are critical variables in the expression of inflammatory signals (34–36) and for this reason we have included both independent variables in the statistical analysis. Here, we show that CCL3 and especially CCL11 were affected by these physiological variables. Regarding CCL3, this is the first time that sex differences are showed in the circulating expression of this chemokine. By contrast, a growing number of clinical studies have described the association of CCL11 with age and sex (37, 38). For example, Targowski and colleagues reported that healthy young subjects (25–34 years) had lower serum levels of CCL11 than older participants (35–55 years). They also showed that there were sex differences with decreased CCL11 concentrations in women (37). Similarly, another study in healthy subjects (40–80 years) demonstrated that the serum levels of CCL11 were gradually increased with age but sex differences were not found, suggesting that sex differences might be abolished with increasing age (38). Our results are in accordance with these studies because we found a significant effect of age on CCL11 concentrations that resulted in a positive correlation, particularly in the control group. As previously mentioned, we observed no differences in CCL11 concentrations in male and female controls but a clear decrease in the expression of this chemokine was found in female compared to male AUD patients. Therefore, alcohol abuse and/or dependence could induce changes in CCL11 concentrations interfering in the expected effects of age and emphasizing sex differences.

### Liver and Pancreas Diseases Influence CXCL8 Concentrations in the Plasma of Abstinent AUD Patients

The influence of variables such as the AUD severity, length of abstinence, and diagnosis of diseases in accessory organs in chemokines was evaluated in the alcohol group. Previously, we have reported an association between plasma concentrations of CXCL12 and CX_3_CL1 and cocaine symptom severity in abstinent cocaine addicts (6) but none of these chemokines were affected by AUD severity. In fact, despite CXCL12 and CX_3_CL1 concentrations were found to be decreased in the alcohol group, none of the alcohol use-related variables had a significant effect. However, alcohol-induced diseases in accessory organs influenced strongly the plasma expression of CXCL8. Thus, the diagnosis of cirrhosis, hepatitis, or pancreatitis that reached about a 24% of AUD patients was associated with elevated CXCL8 concentrations. In line with these data, clinical and preclinical studies have showed that increased concentrations of CXCL8 are involved in the pathogenesis of alcoholic liver and pancreas diseases (39, 40). Predictably, there was an increase of plasma levels of markers linked to alcoholic liver and pancreas damage in patients diagnosed with cirrhosis, hepatitis, and/or pancreatitis. Intriguingly, when we studied the association of these markers with chemokines in our sample, we found only a strong association of AST/GOT and GGT levels with CXCL8 concentrations. Therefore, the present study confirms the role of CXCL8 as a putative indicator of pancreatitis and liver disease.

### Psychiatric Comorbidity Influences CCL11/Eotaxin-1 Concentrations in the Plasma of Abstinent AUD Patients

Most studies on CCL11 are associated with allergy and asthma because this chemokine has been identified as a potential biomarker for the diagnosis and assessment of asthma severity and control (41) but there are no studies on the role of CCL11 in addictive disorders. In our exploratory approach, we studied the potential effect of the presence of psychiatric comorbidity on chemokine concentrations on account of the high rate of prevalence in the alcohol group. As expected, abstinent AUD patients showed an elevated psychiatric comorbidity and about 40% of patients were diagnosed with abuse and/or dependence of other psychoactive substances as well as other mental disorders.

While a pathological use of other substance(s) and alcohol had no effects on any of the chemokines evaluated, the diagnosis of mental disorders had a primary effect on the plasma expression of CCL11. Several studies have reported the association between blood levels of CCL11 and psychiatric disorders, for example in schizophrenia (42), bipolar disorders (43, 44), major depression (45), and psychopathologies with suicidal ideation (45), but there are no studies in a context of substance use disorders. Our data revealed a decreased expression of CCL11 concentrations in abstinent AUD patients diagnosed with non-substance mental disorders. Remarkably, this decrease was observed to be more enhanced in women with mental disorders who displayed lower prevalence of comorbid substance use disorders than men. After evaluating common psychiatric disorders separately, mood disorders and anxiety confirmed their association with lower concentrations of CCL11 in abstinent AUD patients. Evidence points to a strong association between high concentrations in blood CCL11 and the decline in neurogenesis and cognitive impairments (46), and this fact is observed in aged subjects. Accordingly, we have described a clear association between CCL11 concentrations and age but the decreased concentrations observed in comorbid AUD patients are opposite to previous studies in psychiatric disorders, which reported increased concentrations of CCL11 in the serum of patients (7, 42–45). However, none of the referred studies were performed in the context of substance use disorders and several differences are found with respect to the present study.

Interestingly, a clinical study in young adults from general population by Magalhaes and colleagues reported no effects of the diagnosis of substance use disorders as predictor of CCL11 concentrations although both bipolar and depression disorders and sex influenced significantly the expression of this chemokine (7), which is in agreement with the present study in AUD patients. However, the presence of both bipolar and major depression disorders was associated with higher concentrations of CCL11 in contrast to our clinical data. Regarding the association between anxiety disorders and CCL11 concentrations, we found no studies in addicted subjects and only a study in anxious subjects with obsessive–compulsive disorder has examined this chemokine in the plasma but no changes were reported (47). Nevertheless, in the present study we found differences between human and rat. Thus, while anxious AUD patients had lower CCL11 concentrations, ethanol-exposed rats with a previous stress episode by restraint had higher levels of CCL11 in the plasma. It is important to note that all patients were receiving treatment at the time of sampling and the impact that both the pharmacological therapy and the psychological interventions may have on plasma CCL11 concentrations cannot be assessed taking into consideration the cross-sectional design of the present study. Indeed, this potential impact will be studied in future longitudinal studies.

### Limitations and Future Perspectives

The present findings support the importance of monitoring chemokines in the context of complex disorders such as substance use disorders including AUD. We are aware of the limitations of this exploratory study, which need to be borne in mind in interpreting the data. First, the validity of the results presented here needs to be assessed in a larger sample of patients and include psychiatric patients with no history of substance use disorders or pathological use of substances. Second, additional studies to determine plasma chemokines in active alcohol users are necessary to test the effect of alcohol on these inflammatory signals considering the differences and effects observed in preclinical data with rats. Third, the impact of sex differences in both the prevalence of psychiatric disorders and plasma concentrations of chemokines (e.g., CCL3 and CCL11) requires a higher effort on recruiting female participants and a better clinical characterization (stage of menstrual cycle, levels of luteinizing hormone, follicle-stimulating hormone, and estrogen…) because the average age of the women seeking treatment for alcohol problems usually belongs to the menopause transition years. In fact, the lack of data in female rats exposed to ethanol represents a limitation that will need to be addressed in future investigations.

Although CCL11 could be proposed as a potential indicator of psychiatric comorbidity in abstinent subjects with AUD, there are no data about other drugs of abuse. Further clinical and preclinical research is necessary to elucidate the role of chemokines and sex in the etiology of alcoholism and psychiatric disorders.

## Author Contributions

FF, FP, and AS were responsible for the study concept and design. NG-M, GP, PA, MP, and GR coordinated recruitment of participants. NG-M, PA, and MP contributed to the acquisition of psychiatric data by means of interviews and generated the database. AS, EC-O, and JD processed, registered, and stored human blood samples. VB, JC, and JA supervised and performed the quantification of chemokines in human plasma. LS-M, AG, JD, DS, and AS performed rat studies and ELISAs. FP, AS, and PR-S assisted with data analysis and interpretation of findings. FF and FP drafted the manuscript. MT, JA, and GR provided critical revision of the manuscript for important intellectual content. All the authors critically reviewed content and approved the version for publication.

## Conflict of Interest Statement

The authors declare that the research was conducted in the absence of any commercial or financial relationships that could be construed as a potential conflict of interest.

## Abbreviations

ALT/GPT, alanine transaminase; AST/GOT, aspartate transaminase; AUD, alcohol use disorders; CCL11/eotaxin-1, chemokine (C–C motif) ligand 11/eotaxin-1; CCL2/MCP-1, chemokine (C–C motif) ligand 2/monocyte chemotactic protein-1; CCL3/MIP-1α, chemokine (C–C motif) ligand 3/macrophage inflammatory protein-1 alpha; CXCL8/IL-8, chemokine (C–X–C motif) ligand 8/interleukin-8; CXCL12/SDF-1, chemokine (C–X–C motif) ligand 12/stromal cell-derived factor-1; CX_3_CL1/fractalkine, chemokine (C–X_3_–C motif) ligand 1/fractalkine; DSM-IV-TR, Diagnostic and Statistical Manual of Mental Disorders-4th Edition-Text Revision; GGT, gamma-glutamyltransferase; PRISM, Psychiatric Research Interview for Substance and Mental Diseases.
